# A Brief Overview of Uveal Melanoma Treatment Methods with a Focus on the Latest Advances

**DOI:** 10.3390/jcm14124058

**Published:** 2025-06-08

**Authors:** Krystian Wdowiak, Joanna Dolar-Szczasny, Robert Rejdak, Agnieszka Drab, Agnieszka Maciocha

**Affiliations:** 1 University Clinical Hospital No. 4 in Lublin, K. Jaczewskiego 8 St., 20-954 Lublin, Poland; 2 Department of General and Pediatric Ophthalmology, Medical University of Lublin, 20-093 Lublin, Poland; joannaszczasny@op.pl (J.D.-S.); oko@usk1.pl (R.R.); 3 Department of Medical Informatics and Statistics with e-Health Lab, Medical University of Lublin, 20-954 Lublin, Poland; 4 Faculty of Dentistry, Medical University of Lublin, 20-059 Lublin, Poland

**Keywords:** uveal melanoma, darovasertib, treatment, novel therapy

## Abstract

**Background**: Uveal melanoma (UM) is a relatively rare malignancy, yet it remains the most common primary intraocular cancer in adults. Several risk factors have been identified, including light iris color, fair skin tone, and cutaneous freckles. **Methods**: The aim of this article was an overview of the treatment methods for uveal melanoma, with a particular focus on emerging therapies such as tebentafusp and da-rovasertib. The research method was a review of the latest literature. **Results**: Genetic studies have uncovered key mutations in *GNAQ* and *GNA11*, which significantly contribute to UM pathogenesis. Treatment selection depends on tumor location and disease stage. In localized disease, radiotherapy—especially brachytherapy—is commonly used and generally effective. However, the prognosis worsens significantly once distant metastases, most often to the liver, develop, as no standard systemic therapy has demonstrated high efficacy in this setting. Recent years have seen the emergence of promising therapies, including tebentafusp, which stimulates immune responses against gp100-expressing melanoma cells, and darovasertib, a potent PKC inhibitor that targets MAPK pathway activation driven by *GNAQ*/*GNA11* mutations. Both agents have shown encouraging tolerability; tebentafusp has demonstrated clinical benefit in Phase II and III trials, while darovasertib is still under investigation. Additionally, melphalan-based liver-directed therapy, particularly via hepatic arterial infusion (approved by the FDA), has shown potential in controlling liver-dominant disease in metastatic UM. This localized approach may provide significant benefit for patients with limited extrahepatic spread. **Conclusions**: Future research should focus on optimizing these novel strategies—tebentafusp, darovasertib, melphalan, and combination therapies—and on expanding our understanding of UM’s molecular drivers to enable the development of more effective, personalized treatments.

## 1. Introduction

Melanoma is a malignant tumor originating from melanocytes, most commonly found in the skin. However, it can also develop in the mucous membranes and the eye [1]. Ocular melanoma (OM) is a rare malignant tumor, with only a few to a dozen cases diagnosed per million people in a given population each year [2,3,4]. A review conducted by Weinberger et al. [4,5] of data available from the Surveillance, Epidemiology, and End Results (SEER) Program of the National Cancer Institute, covering the period from 1975 to 2020, showed that the age-adjusted incidence of this tumor was 5.6 per million.

Ocular melanoma can be broadly categorized into two major groups: uveal melanoma (affecting the choroid, ciliary body, and iris) and non-uveal melanoma (affecting the conjunctiva and other ocular locations) [3]. Uveal melanoma (UM) accounts for the vast majority of OM cases, representing approximately 84.5% of all cases of this malignancy [4,5]. The most common site of UM is the choroid (71.3% of all OM cases), while tumors located in the ciliary body and iris account for 12.2% of OM cases [4,5]. As for OM located outside the uvea, the conjunctiva is the most frequent site, comprising 6.9% of all cases. Despite its relatively rare occurrence, uveal melanoma is the most common intraocular malignancy in adults. In contrast, retinoblastoma is the most prevalent intraocular malignant tumor in children [6,7].

Over the past years, research has identified several risk factors for UM, particularly those associated with pigmentation. These include iris color, fair skin tone, tanning ability, atypical and common cutaneous nevi, cutaneous freckles, and iris nevi [8,9,10]. The influence of UV radiation on UM development has been the subject of numerous studies in recent years [11,12] and remains a complex issue. The incidence of choroidal and ciliary body UM tends to increase with lower UV exposure, which contrasts with iris melanoma. It is worth noting that OM located in the eyelid and conjunctiva is more frequently observed in areas with higher UV radiation exposure [11]. The described impact of UV radiation on the incidence of different OM types appears to be the result of its varying effects on carcinogenesis—stimulating it in some cases while inhibiting it in others [11,12]. As can be observed, many risk factors for UM overlap with those of cutaneous melanoma (CM). Nevertheless, an important distinction is that UM typically develops due to mutations in the *GNAQ* or *GNA11* genes, which differ from the genetic basis of cutaneous melanoma [6].

It is also worth mentioning the results of a relatively recent study conducted by Salzano et al. [13], which examined S100 immunoreactivity in primary uveal melanoma (UM), metastatic UM, and metastatic cutaneous melanoma (CM). The study found that S100 immunoreactivity is common in metastatic CM and primary UM, but rare in metastatic UM [13]. This finding may aid in distinguishing the origin of melanoma metastases, particularly in cases where the primary tumor site is unknown.

A comparison between uveal and cutaneous melanoma is presented in Table 1.

Advancements in genetic research have also enabled the identification of several single-nucleotide polymorphisms (SNPs) associated with an increased risk of UM. Among these are rs3759710, rs421284, rs12913832, and rs12203592 [14,15].

Uveal melanoma is a malignancy with a poor prognosis, frequently leading to distant metastases, particularly in the liver, lungs, and bones [16,17]. The treatment of UM varies depending on the stage of disease progression, with different therapeutic approaches being employed accordingly [18].

In this article, we provide a brief overview of the treatment methods for uveal melanoma, with a particular focus on emerging therapies such as tebentafusp and darovasertib.

## 2. Established Treatment Methods for Uveal Melanoma

**Figure 1:**
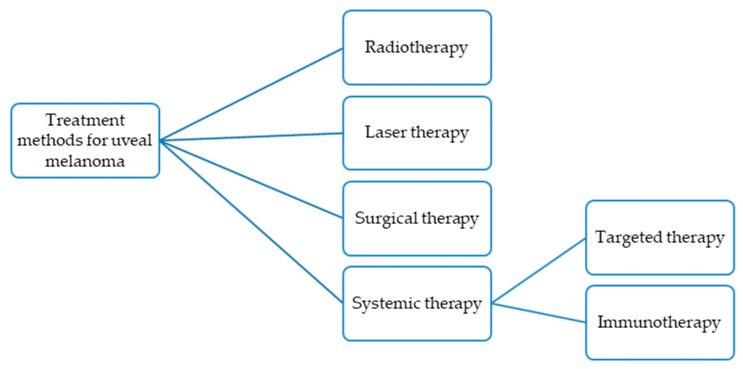
Established treatment methods for uveal melanoma.

Current treatment strategies for uveal melanoma incorporate various techniques, including radiotherapy, laser therapy, surgical interventions, and systemic therapy (Figure 1), [18,19].

Radiotherapy is a vision-preserving treatment modality that can be used as a standalone therapy or as an adjunctive treatment [20,21]. One of the most widely used radiotherapy techniques for uveal melanoma is brachytherapy, which involves suturing a radioactive plaque containing a radioisotope (most commonly I-125 or Ru-106) onto the sclera near the tumor. The plaque remains in place for a few days before being removed [18,19,22,23]. Recent studies have demonstrated the high efficacy of this approach, showing no significant difference in outcomes compared to surgical treatments for medium-sized tumors [18]. Another radiotherapy approach used in the treatment of uveal melanoma is charged particle radiation therapy, in which charged particles (such as protons, helium ions, or carbon ions) are precisely directed at the tumor tissue. This technique allows for highly targeted delivery of radiation, thereby minimizing damage to surrounding healthy structures [18,19,24,25,26]. Researchers have highlighted its particular utility in cases involving larger tumors or those located near the optic nerve [18,19,24]. A meta-analysis conducted a few years ago [25] comparing brachytherapy and charged particle radiation therapy found that the latter was associated with a lower rate of local recurrence. However, there were no significant differences between the two methods in terms of mortality or enucleation rates. Additionally, charged particle therapy has been associated with lower rates of radiation-induced retinopathy and cataract formation compared to brachytherapy [25]. Stereotactic radiosurgery (SRS) is also used in the management of uveal melanoma. This technique enables the precise delivery of high-dose radiation to tumor tissues, but it requires high-resolution imaging to accurately delineate tumor margins. As a result, SRS is typically applied in the treatment of larger lesions [26]. While SRS generally provides a satisfactory therapeutic outcome, it is often linked to vascular complications, including secondary glaucoma.

Historically, photocoagulation techniques were used in the treatment of uveal melanoma; however, due to their high rate of complications and limited therapeutic success, they are now rarely applied [18,27]. Modern phototherapy techniques primarily include photodynamic therapy and transpupillary thermotherapy. These methods are mainly used as neoadjuvant therapies or as adjuvant treatments following radiotherapy or surgical intervention [18,28,29].

Surgical interventions for uveal melanoma can generally be categorized into two main groups: local resection techniques, such as iridectomy, iridocyclectomy, exoresection, and endoresection, and radical procedures, including enucleation and exenteration. These surgical methods are primarily reserved for cases where the tumor is too large or its location limits the feasibility of radiotherapy [18]. The selection of the appropriate local surgical technique largely depends on the tumor’s size and precise location. For iris melanoma, procedures such as iridectomy and iridocyclectomy are commonly performed. In specific cases of iridociliary or choroidal melanomas, exoresection may be considered a viable treatment option. When the tumor is positioned posteriorly, near critical structures such as the optic papilla or macula, endoresection is typically preferred [18,30,31,32,33]. For cases of locally advanced uveal melanoma that cannot be effectively managed with other therapeutic modalities, enucleation becomes necessary. In instances where the tumor has infiltrated surrounding tissues, a more extensive procedure known as exenteration may be required [18]. While these surgical treatments have proven to be effective in many cases of uveal melanoma, their success remains contingent on the absence of distant metastases. Unfortunately, once metastatic spread occurs, treatment success rates decline significantly. However, ongoing advancements in novel therapeutic approaches hold the potential to improve these outcomes in the coming years [18,19].

Systemic treatment strategies for uveal melanoma have been classified into two primary categories: targeted therapy and immunotherapy. Targeted therapy involves the use of pharmacological agents that specifically influence cancer cells by disrupting key signaling pathways or interfering with molecules secreted by tumor cells. Previous studies have highlighted the hyperactivation of tyrosine kinases in uveal melanoma, attributed to the overexpression of the c-KIT protein. As a result, efforts have been made to explore tyrosine kinase inhibitors as potential treatment options for this malignancy [34,35,36,37]. Investigations have been conducted on agents such as imatinib mesylate, sunitinib, and crizotinib, with sunitinib demonstrating the most promising therapeutic outcomes among them [34,35,36,37]. Vascular endothelial growth factor (VEGF) is known to be overexpressed in various malignancies, contributing to tumor progression and metastasis. This phenomenon has also been observed in uveal melanoma, where elevated VEGF levels in the aqueous humor have been correlated with tumor size [18]. In light of this, VEGF inhibitors such as bevacizumab, ranibizumab, and sorafenib have been investigated as potential treatments for uveal melanoma [38,39,40,41]. While these agents show promise, further studies are required to evaluate their long-term efficacy and mitigate adverse effects. Nonetheless, they are likely to play a crucial role in adjuvant therapy [18]. As previously mentioned, the development of uveal melanoma is frequently associated with mutations in the *GNAQ* and *GNA11* genes, which drive excessive activation of the mitogen-activated protein kinase (MAPK) pathway [18]. Selumetinib, a drug targeting this signaling cascade (as it is an inhibitor of MAPK subtype 1 and 2), has been evaluated for its potential to enhance the efficacy of chemotherapy in the treatment of uveal melanoma [42,43].

Similar to cutaneous melanoma, immunotherapy has been explored as a systemic treatment option for uveal melanoma. Unfortunately, the application of immunotherapy in uveal melanoma has often failed to produce satisfactory results [18]. This phenomenon can be attributed, among other factors, to differences in mutational burden between these two malignancies—in cutaneous melanoma the mutational burden is typically high, whereas in uveal melanoma it is lower. This correlates with the number of neoantigens produced, which can be recognized by the immune system [18]. Another reason for the lower efficacy of immunotherapy in uveal melanoma is the immune-privileged status of the eye, which naturally suppresses immune responses within this organ [18]. Initial investigations focused on interferon (IFN)-α-2a, though the clinical outcomes were largely unsatisfactory [18]. Subsequent research efforts shifted towards the use of ipilimumab, a human cytotoxic T-lymphocyte-associated antigen 4 (CTLA-4) inhibitor, which enhances the cytotoxic activity of T cells against tumor cells [44,45,46]. Phase II studies conducted in recent years on combination therapy with nivolumab and ipilimumab have provided encouraging data regarding its efficacy—the therapy had a positive impact on 3-year distant metastasis-free survival, which differed significantly from the control group [45,46].

A brief overview of the application of various treatment methods for uveal melanoma is presented in Table 2.

## 3. Tebentafusp

Tebentafusp belongs to the group of immune-mobilizing monoclonal T-cell receptor (TCR) therapies targeting cancer. It is a molecule with high affinity for gp100, a protein abundantly presented by HLA-A*02:01 on melanoma cells, as well as for the CD3 receptor expressed on T lymphocytes [47,48]. The mechanism of action of tebentafusp involves significant activation of the immune system, leading to strong stimulation of both Th and Tc lymphocytes. This, in turn, results in an intensified cytotoxic response directed against tumor cells, with the magnitude of the response increasing in correlation with the expression of gp100 on the tumor surface [49].

In 2022, tebentafusp received approval from the U.S. Food and Drug Administration (FDA) for the treatment of metastatic uveal melanoma (UM) [50,51]. Phase I/II clinical trials demonstrated good tolerability of tebentafusp-based therapy [52,53].

In the referenced Phase II study, the overall response rate (ORR) was 5%, a reduction in target lesions was observed in 44% of patients, and the 12-month overall survival rate ranged from 62% to 86% [53].

The subsequent Phase III trial showed a significantly increased 12-month overall survival rate in patients treated with tebentafusp compared to the control group (73% vs. 59%), as well as a significantly improved progression-free survival (31% vs. 19% at 6 months) [54]. Taken together, these results indicate a positive impact of tebentafusp on overall survival in metastatic UM.

## 4. Darovasertib

Genetic studies conducted on histological samples obtained from patients diagnosed with uveal melanoma (UM) have revealed that in more than 90% of cases, activating mutations can be detected in the *GNAQ* and *GNA11* genes, which encode the Gq alpha subunits [54]. The majority of these mutations occur at codon 209 of the Gq alpha subunit, leading to the loss of intrinsic GTPase activity. As a result, this mutation causes constitutive activation of the MAPK signaling pathway, which plays a crucial role in UM tumor progression [54]. One of the strategies to counteract the overactivation of the MAPK pathway is the inhibition of protein kinase C (PKC), a key downstream effector in this signaling cascade [54,55].

**Figure 2:**
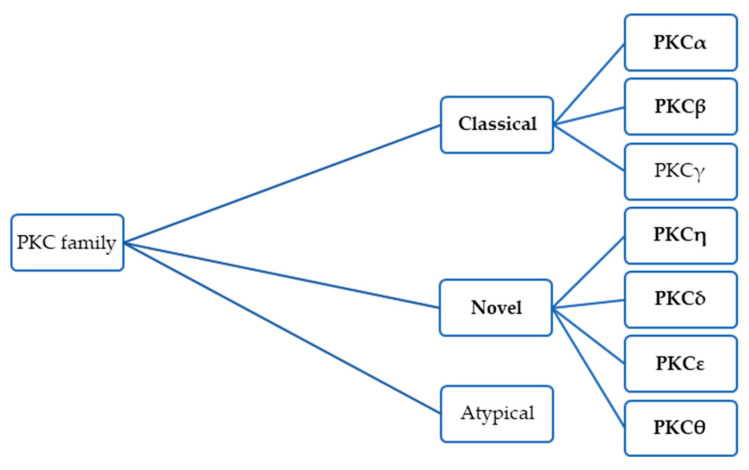
The PKC family, with an indication of the target sites of darovasertib.

The PKC family consists of at least twelve isoenzymes, which have been classified into three groups based on their activation mechanisms: classical PKCs (PKCα, two isoforms of PKCβ, and PKCγ), novel PKCs (PKCη, PKCδ, PKCε, and PKCθ), and atypical PKCs (PKCζ, PKCι, PKCμ, and PKCν) [54]. Among these, the classical and novel PKC isoforms play a particularly important role in UM pathogenesis. Figure 2 shows the PKC family, with an indication of the target sites of darovasertib.

Darovasertib is the first orally administered and highly potent PKC inhibitor that selectively targets isoforms from both classical and novel PKC groups, with greater selectivity toward novel PKC isoforms. This includes PKCα, PKCβ, PKCη, PKCδ, PKCε, and PKCθ, making it a promising therapeutic agent for the treatment of uveal melanoma [56,57,58,59].

Darovasertib was granted orphan drug status by the U.S. Food and Drug Administration (FDA) in 2022 [56].

In recent years, a Phase I clinical trial was conducted to evaluate the use of darovasertib as monotherapy. The study included 68 patients with metastatic uveal melanoma (UM) and demonstrated satisfactory treatment tolerability. In terms of efficacy, clinical activity was observed in 9.1% of cases, while 68% of patients achieved stable disease [60,61]. However, it is important to note that the primary goal of Phase I trials is to assess drug safety, and efficacy-related results should be interpreted with caution and confirmed in subsequent studies.

A Phase II trial is currently underway, which so far provides encouraging data on the potential efficacy of darovasertib in neoadjuvant/adjuvant treatment of localized primary UM [62].

In recent years, studies have also emerged aiming to assess the safety and efficacy of combination therapy using darovasertib in conjunction with crizotinib (a MET and ALK kinase inhibitor) [63,64,65,66]. So far, these findings are mainly limited to in vitro conditions, animal models, and case reports, and should therefore be interpreted with caution. It is also worth noting the ongoing Phase II/III clinical trial (NCT05987332), which aims to evaluate the efficacy of this drug combination [67,68].

In summary, darovasertib—both as monotherapy and in combination with crizotinib—appears to be a safe treatment option for UM; however, its efficacy still requires further evaluation, which will be addressed in the ongoing Phase II/III trials.

## 5. Melphalan

As previously mentioned, distant metastases are common in uveal melanoma (UM), with the liver being the most frequent site—occurring in up to 90% of cases according to some sources—and often leading to hepatic failure [69]. Given this high prevalence, the development of effective treatment strategies for UM liver metastases remains a critical clinical challenge.

Among the available treatment options—many of which are also used for liver metastases from other cancers—are transarterial chemoembolization (TACE) and radioembolization [70]. TACE is a widely used technique for treating hepatic tumors. It involves delivering cytotoxic drugs mixed with lipiodol, which acts both as a drug carrier and an embolic agent (conventional TACE, or cTACE), or using drug-eluting beads (DEB-TACE) [70]. While most clinical data on TACE come from studies on hepatocellular carcinoma, in recent years, its application in treating UM liver metastases has also been explored. Its effectiveness, however, depends largely on the tumor’s vascularity and especially its size [70].

Radioembolization, on the other hand, involves the selective delivery of yttrium-90 (^90^Y)-labeled microspheres into the hepatic artery. Due to the limited tissue penetration of the emitted radiation, this technique allows for targeted tumor destruction while sparing surrounding healthy liver tissue [70]. This method has been widely used for both primary and metastatic liver tumors. However, its use in UM is relatively recent and, according to several studies, appears to be a promising therapeutic option [70].

Lastly, it is worth mentioning a relatively new FDA-approved treatment for UM liver metastases involving the administration of melphalan [71]. Melphalan is a bifunctional alkylating agent that binds to the N7 position of guanine, causing inter- and intrastrand DNA cross-links and inducing cytotoxic effects, thereby exerting its anticancer properties [71,72]. The drug is also used in the treatment of various malignancies, including multiple myeloma, ovarian adenocarcinoma, breast cancer, and neuroblastoma [72].

Studies investigating the use of melphalan in the treatment of liver-metastatic UM have employed two delivery techniques: isolated hepatic perfusion (IHP) and percutaneous hepatic perfusion (PHP) [73,74]. IHP is an open surgical procedure designed to separate the liver from the systemic circulation, allowing for the administration of higher doses of chemotherapeutic agents without affecting the rest of the body [73]. In contrast, PHP is a less invasive, endovascular technique in which the liver is temporarily isolated from systemic circulation and perfused with chemotherapy delivered directly into the hepatic artery [74].

In recent years, phase III clinical trials have been conducted to assess the safety and efficacy of both approaches, demonstrating clinically meaningful responses. A meta-analysis comparing the two methods has also been performed [73,74,75]. The findings suggest that both techniques offer comparable efficacy, as indicated by similar median overall survival and median progression-free survival. However, PHP is associated with a lower risk of complications [75].

## 6. Conclusions

Despite its relatively rare occurrence, uveal melanoma (UM) remains a significant challenge for healthcare systems. Over the past few years, considerable progress has been made in the treatment of this malignancy. However, therapeutic success in advanced stages of the disease remains unsatisfactory, despite ongoing efforts to introduce new therapeutic agents.

Expanding our understanding of the genetic and molecular characteristics of UM is crucial. While UM shares some similarities with cutaneous melanoma, it also exhibits distinct features that may be leveraged for more targeted treatment strategies. Notably, the identification of *GNAQ* and *GNA11* mutations has provided valuable insights into the disease’s pathogenesis.

In recent years, several new potential therapeutic options for uveal melanoma (UM) have emerged, most notably the use of tebentafusp or darovasertib. Tebentafusp, an immune-mobilizing monoclonal TCR therapy, enhances cytotoxic immune responses against melanoma cells by targeting gp100, which is highly expressed on their surface. Meanwhile, darovasertib, a potent protein kinase C (PKC) inhibitor, disrupts oncogenic signaling within the mitogen-activated protein kinase (MAPK) pathway, which is frequently activated due to *GNAQ* and *GNA11* mutations.

Studies conducted to date indicate satisfactory safety of tebentafusp in the treatment of UM. Phase III trials have demonstrated a positive impact of this drug on overall survival in metastatic UM.

Current research also suggests that darovasertib is well tolerated in the treatment of UM; however, objective conclusions regarding its efficacy can only be drawn after the completion of the ongoing clinical trials.

Combination therapies are also being explored, including the use of darovasertib with crizotinib. More definitive data on the effectiveness of this therapeutic approach are expected upon the completion of the ongoing Phase II/III trials.

Future research should focus on further evaluating recently developed treatment strategies for uveal melanoma—such as darovasertib, tebentafusp, and the combination therapies mentioned in this article—as well as therapies specifically targeting metastases in certain organs (e.g., melphalan for liver metastases). Continued investigation into the genetic basis of uveal melanoma may also lead to the development of effective, targeted therapies.

## Figures and Tables

**Table 1:** Comparison of various characteristics of cutaneous and uveal melanoma [1,6,13].

Characteristic	Cutaneous Melanoma	Uveal Melanoma
Incidence rate	12.2 to 48.1 cases per 100,000 individuals	5.6 cases per million individuals
Typical age at diagnosis	Generally younger, with a median age of 57 years	Generally older, with peak incidence at 70–75 years
Prevalence among melanomas	Around 90% of all primary melanoma cases	Around 5% of all primary melanoma cases
Association with UV exposure	Strongly linked to UV radiation	Varies depending on tumor location
Common genetic mutations	Typically involves mutations in *BRAF* or *NRAS* genes	Most often involves *GNAQ* or *GNA11* gene mutations
S100 immunoreactivity	Common in metastatic CM	Common in primary UM, rare in metastatic UM
5-year survival rate	~94%; ~32% in metastatic disease	~85%; ~15% in metastatic disease

**Table 2:** Brief summary of the use of established treatment methods for uveal melanoma.

Treatment Modality	Indications	Efficacy	Complications
Brachytherapy	Small to medium-sized tumors; vision-sparing approach	High local tumor control; outcomes comparable to surgery for medium-sized tumors	Radiation retinopathy, optic neuropathy, cataract
Charged particle therapy	Larger tumors or tumors near optic nerve	High precision and effective local control	Limited availability; radiation damage to nearby critical structures
Stereotactic radiotherapy	Select tumors unsuitable for other radiotherapy approaches	Effective in selected cases	Possible collateral tissue damage
Photodynamic therapy/transpupillary thermotherapy	Mainly used as neoadjuvant/adjuvant therapy	Limited as monotherapy; can improve outcomes when combined with other treatments	Retinal damage, limited penetration in deeper tumors
Local resection	Small to medium-sized tumors	Organ-sparing; variable success based on tumor location and surgeon experience	Risk of recurrence; surgical complications
Enucleation	Large tumors, advanced disease	Definitive local control	Loss of eye; psychological impact
Exenteration	Orbital invasion by tumor	Only option in very advanced cases	Highly disfiguring; functional and psychological burden
Systemic therapy (targeted & immunotherapy)	Metastatic or unresectable disease; mutation- or immune-guided therapy	Generally limited efficacy; combination immunotherapy and targeted agents shows some promise	Systemic toxicity, immune-related adverse events, incomplete or short-lived response

## Data Availability

Data are contained within the article.
